# Acupuncture Regulating Gut Microbiota in Abdominal Obese Rats Induced by High-Fat Diet

**DOI:** 10.1155/2019/4958294

**Published:** 2019-06-02

**Authors:** Haiying Wang, Qiang Wang, Cuimei Liang, Mingxing Su, Xin Wang, Hua Li, Hui Hu, Hongjuan Fang

**Affiliations:** ^1^Dong Fang Hospital, Beijing University of Chinese Medicine, Beijing 100078, China; ^2^Institute of Disease Control and Prevention of Chinese People's Liberation Army, Beijing 100071, China; ^3^Beijing Hospital of Traditional Chinese Medicine Affiliated to Capital Medical University, Beijing 100010, China; ^4^Department of Endocrinology, Beijing Tiantan Hospital, Capital Medical University, Beijing 100070, China

## Abstract

**Objective:**

To investigate the effects of acupuncture on metabolic health and gut microbiota dysbiosis in diet-induced abdominal obese model.

**Materials and Methods:**

Male Sprague-Dawley rats were randomly distributed into normal chow diet (NCD) group and high-fat diet (HFD) group. After 12 weeks of HFD feeding, an abdominal obese rat model was established. The abdominal obese rats were further assigned to acupuncture group (n=7) and nontreated HFD group (n=7). Acupuncture was applied to bilateral GB 26 of rats for 8 weeks. Subsequently, the body weight, waist circumference (WC), visceral fat mass, and liver weight were measured weekly in all rats. Metabolic parameters such as total cholesterol, triglyceride, alanine aminotransferase, aspartate transaminase, and blood glucose were measured by an automatic biochemical analyzer. The serum levels of insulin (INS) were determined using Rat INS ELISA Kit. Analysis of gut microbiota was carried out by 16S rRNA gene sequencing.

**Results:**

Acupuncture decreased the body weight, WC, and visceral adipose tissues of HFD-induced abdominal obese rats. In addition, insulin sensitivity, glucose homeostasis, and lipid metabolism were improved by this treatment. Furthermore, electroacupuncture effectively modified the composition of gut microbiota, mainly via decreasing Firmicutes/Bacteroidetes ratio and increasing Prevotella_9 abundance.

**Conclusions:**

Electroacupuncture can ameliorate abdominal obesity and prevent metabolic disorders in HFD-induced abdominal obese rats, via the modulation of gut microbiota.

## 1. Introduction

Obesity is a multifactorial disease resulting from the interplay between environmental, cultural, behavioral, metabolic, and genetic factors [1–5]. Compared with general obesity, abdominal obesity is considered to be more closely associated with the risks of metabolic disease [6, 7], nonalcoholic fatty liver disease [8, 9], and cardiovascular diseases [10, 11], which ranks as an independent risk factor of all-cause mortality [12]. It has been reported that the human microbiota encompasses trillions of microbes residing in the gut [13, 14]. Recent studies have found that the structure, functionality, and diversity of gut microbiota may play a pivotal role in the development and progression of obesity [15–18]. Increasing evidence indicates that an aberrant Firmicutes/Bacteroidetes ratio is associated with the risk of overweight and obesity [19–22]. Therefore, a deeper understanding of how gut microbiota dysbiosis can help to elucidate the pathogenesis of obesity and potentiate a novel therapeutic approach for obesity-related comorbidities.

Recently, there appears to be a growing interest among researchers on the role of acupuncture in disease prevention and treatment. Some animal studies have found that acupuncture can exert significant effects on the treatment of obesity [23–25]. Acupuncture, a time-honored tradition in Chinese Medicine, has been proven to be an effective weight-loss medication. Besides, the 26^th^ acupuncture point of the Gall Bladder Meridian (GB26), which located on the lower abdomen, is possibly associated with the accumulation of abdominal adipose tissues [26, 27]. Given all these considerations, we sought to investigate the correlation between the gut microbiota and abdominal obesity, as well as the effects of acupuncture on metabolic health and gut microbiota dysbiosis in diet-induced obesity model.

## 2. Materials and Methods

### 2.1. Animals

Thirty seven specific pathogen-free male Sprague-Dawley (SD) rats (5-6 weeks old, 220±20g) were obtained from the Experimental Animal Center of Military Medical Science Academy (license number: SCXK (Jun) 2017–00004). Rats were housed 3-4 per cage, under controlled environmental conditions (12 h:12 h light:dark cycle, 22±2°C room temperature, and 50-60% relative humidity). They were given ad libitum access to food and water. The necessary steps were taken to minimize pain and distress in these animals. All experimental procedures were in strict accordance with the animal welfare principles governed by the Animal Ethics Committee of the Institute of Disease Control and Prevention of the Chinese People's Liberation Army.

### 2.2. Materials and Reagents

The sterile and single-used acupuncture needles (0.30 mm×25 mm) were purchased from Beijing Zhongyan Taihe Medical Instrument Co., Ltd (Beijing, China), whereas electro pulse acupuncture therapeutic apparatus (YINGDI KWD-808) was obtained from Changzhou Yingdi Electronic Medical Devices Co., Ltd. (Jiangsu, China). The high-fat diet (HFD; 2.5% cholesterol, 0.3% sodium cholate, 20% sucrose, 20% lard, and 57.2% basic feed) was supplied from Beijing Botai Hongda Biotechnology Co., Ltd., while the normal chow diet (NCD; 24% corn flour, 20% bran, 20% bean cake, 20% flour, 6% cellulose, 5% fish meal, 3% bone meal, and 2% salt) was provided by the Experiment Animal Center of Military Medical Science Academy. Laboratory equipment such as electronic balance (AL 204, METTLER TOLEDO, Switzerland), automatic biochemical analyzer (MEK7222K, Nihon Kohden, Japan), Illumina MiSeq platform (San Diego, USA), and NanoDrop 2000 UV-vis spectrophotometer (Thermo Scientific, Wilmington, USA) was used throughout the experiments. Rat Insulin (INS) ELISA Kit (Catalogue Number: SU-B30620) and 10% chloral hydrate (batch number: 20150303) were obtained from the Sinopharm Chemical Reagent Co., Ltd. (Beijing, China). E.Z.N.A.® soil DNA Kit was purchased from Omega Bio-tek (Norcross, GA, USA).

### 2.3. Establishment of an Abdominal Obesity Rat Model

After a week of acclimatization, all the 37 male SD rats were randomly divided into 2 groups: NCD group (n=7) and HFD group (n=30). Their body weight and waist circumference (WC) were measured weekly during the 12-week feeding period. Abdominal obese rats were identified based on their body weight (20% higher than normal weight) [28] and WC (significantly different compared to those in NCD group) [29]. Finally, 14 abdominal obese rats were randomly allocated into two groups: HFD model group (n=7) and acupuncture treatment group (n=7). These 14 rats were continuously fed with HFD, while the other 7 rats in NCD group were continued with NCD feeding.

### 2.4. Acupuncture Treatment

To eliminate the restraint stress, the rats in acupuncture group were fixed in rat pockets before acupuncture treatment, and the rats of HFD group were also fixed in rat pockets at the same time. Meanwhile, the rats in NCD group received no intervention. After that, sterile acupuncture needles (0.30 mm×25 mm) were inserted vertically into the bilateral “Daimai” (GB 26) acupoint with a depth of 4-5 mm. Subsequently, electroacupuncture was applied to the same acupoint using YINGDI KWD-808 electro pulse acupuncture therapeutic apparatus at a frequency of 2 Hz/15 Hz and an intensity of 1.5 mA for 20 minutes. Treatments were carried out on alternate weekdays (Monday/Wednesday/Friday) for 8 weeks. All rats were given ad libitum access to food and water, and their body weight and WC were measured once a week.

### 2.5. Measurement of Body Weight, WC, Food Intake, and Adipose Tissues

The body weight, WC (mid-point of the perpendicular distance between abdominal xiphoid process and hind legs), and food intake were measured weekly using an electronic scale and a nonelastic measuring tape, respectively. After 8 weeks of acupuncture treatment, all rats were deprived of food overnight (12 hours), followed by anesthesia induction via an intraperitoneal injection of 10% chloral hydrate (3 mL/kg). Their liver and adipose tissues including those around the kidneys and near the epididymis were weighed. Adiposity index was calculated by the following formula: 100 × (the weights of adipose tissues around the kidneys and near the epididymis)/body weight. Meanwhile, liver index was calculated by the following formula: (liver weight/body weight) × 100.

### 2.6. Metabolic Parameters

Animals were anesthetized by intraperitoneal injection with 10% chloral hydrate (3 mL/kg). Blood samples were collected from the abdominal aorta of rats and then centrifuged at 3000 r/min, for 10 min, at 4°C. Following centrifugation, the separated serum was collected and stored at −80°C until further analysis. The serum levels of triglycerides (TG), total cholesterol (TC), alanine aminotransferase (ALT), aspartate aminotransferase (AST), and blood glucose (GLU) were analyzed by an automatic biochemical analyzer. The levels of FINS were measured by Rat INS ELISA Kit, and the index of homeostasis assessment of insulin resistance (HOMA-IR) was calculated by the following formula: HOMA-IR = fasting blood glucose (FBG) × fasting blood insulin (FINS)/22.5.

### 2.7. DNA Extraction and 16S rRNA Gene Amplification and Sequencing

After anesthesia, fecal samples were obtained from the three groups and immediately stored at −80°C for 16S rRNA sequencing. Microbial DNA was extracted from the fecal samples using E.Z.N.A.® soil DNA Kit. The final concentration and purity of DNA were determined by NanoDrop 2000 UV-vis spectrophotometer, and the quality of DNA was evaluated with 1% agarose gel electrophoresis. The hypervariable regions (V3-V4) of the bacterial 16S ribosomal RNA (rRNA) gene were amplified with a set of primers (338F: 5′-ACTCCTACGGGAGGCAGCAG-3′ and 806R: 5′-GGACTACHVGGGTWTCTAAT-3′). Polymerase chain reaction (PCR) conditions were as follows: an initial denaturation step at 95°C for 2 min, followed by 25 cycles of denaturation at 95°C for 30s, annealing at 55°C for 30s and extension at 72°C for 30s, and a final extension step at 72°C for 5 min. The PCR amplification was carried out in a GeneAmp PCR System 9700 thermo cycler (Applied Biosystems, Foster City, CA, USA). After PCR, amplicons are purified and pooled in equimolar and paired-end sequenced (2×300) on an Illumina MiSeq platform, according to the instructions of Majorbio Bio-Pharm Technology Co., Ltd. (Shanghai, China).

Quality control for raw sequencing sequence with Trimmomatic software and stitching using FLASH software were as follows: (1) Set up a 50bp window. If the average quality value within the window falls lower than 20, truncate the back base from the window and then remove the sequence shorter than 50 bp during the quality control; (2) match barcodes exactly, allow mismatches up to 2 bases for primers, and remove ambiguous bases; (3) stitch the sequences of the two ends according to the overlapping base overlap, with the overlap being greater than 10bp. Remove the sequences that cannot be stitched [30].

### 2.8. Bioinformatics and Statistical Analysis

UPARSE version 7.1 (http://drive5.com/uparse/) was used to cluster the operational taxonomic units (OTUs) with 97% similarity cutoff, while chimeric sequences were identified and removed by UCHIME. The taxonomy of each 16S rRNA gene sequence was constructed by RDP Classifier algorithm (http://rdp.cme.msu.edu/) against the Silva (SSU123) 16S rRNA database, with a confidence threshold of 70% [30]. Rarefication curve was analyzed on the normalized OTU level using R (verson3.3.1), and Alpha diversity (sobs index and ace index) and beta diversity were calculated using QIIME [31, 32]. The representative sequences of OTUs obtained from rarefication were used to generate a phylogenetic tree using FasTree and the Distance Algorithm of Principal coordinates analysis (PCoA), and Hierarchical clustering was used for unweighted UniFrac. The differences between gut microbiota in the three groups were calculated by Bray-Curtis distances and Unweighted Pair-group Method with Arithmetic Mean (UPGMA) and visualized using PCoA and Hierarchical clustering at the OUT level. Statistical analysis was performed using SPSS version 20.0 (IBM Corp., Armonk, NY, USA). Multiple comparisons between groups were analyzed by one-way ANOVA followed by Least-Significant Difference (LSD) test, or otherwise a nonparametric Kruskal-Wallis test was used if the data did not meet the assumptions of t-test or one-way ANOVA. All data were expressed as mean ± standard deviation (SD).* P* values of less than 0.05 were regarded as statistically significant.

## 3. Results

### 3.1. Effects of Acupuncture on Body Weight, WC, Visceral Fat Mass, Liver Mass, and the Indices of Liver and Adiposity

**Figure 1:**
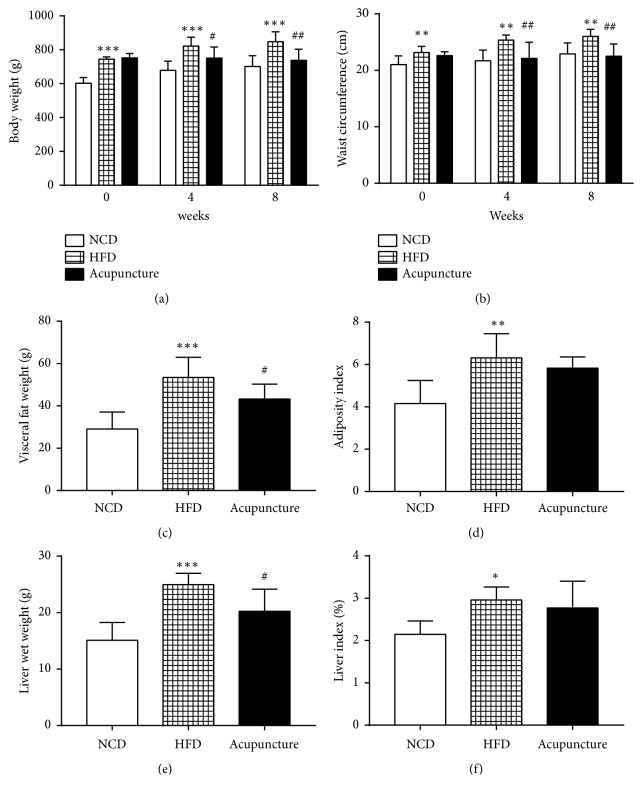
Antiobesity effects of acupuncture on HFD-induced abdominal obese rats. (a) Changes in body weight among NCD, HFD, and acupuncture groups (n=7 in each group) at weeks 0, 4, and 8. (b) Changes in waist circumference among the three groups at weeks 0, 4, and 8. (c) Visceral fat accumulation (adipose tissues around the kidneys and near the epididymis). (d) The values of adiposity index. (e) The weight of liver. (f) The values of liver index. All data are expressed as mean ± SD. ^∗^*P* < 0.05, ^∗∗^*P* < 0.01, ^∗∗∗^*P* < 0.001, NCD vs. HFD; ^#^*P* < 0.05, ^##^*P* < 0.01, HFD vs. acupuncture.

**Figure 2:**
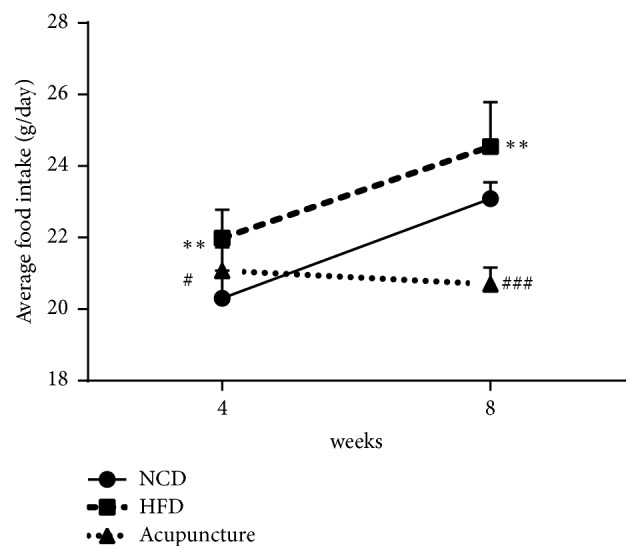
Average food intake (g/day) in the three groups. ^∗∗^*P* < 0.01, NCD vs. HFD; ^#^*P* < 0.05, ^##^*P* < 0.01, HFD vs. acupuncture.

At week 0, the body weight (*P *< 0.001) and WC (*P *< 0.01) of HFD rats were significantly higher than those of NCD rats (Figures 1(a)-1(b)). However, there were no significant differences (*P* > 0.05) in the body weight and WC between HFD and acupuncture groups (Figures 1(a)-1(b)). At week 8, the rats in HFD group displayed increased body weight (*P *< 0.001), WC (*P *< 0.01), visceral fat mass (*P* < 0.001), liver weight (*P* < 0.001), adiposity index (*P *< 0.01), and liver index (*P *< 0.05), compared to NCD group (Figures 1(a)–1(f)). On the other hand, the rats in acupuncture group demonstrated lower body weight (*P *< 0.01) and WC (*P *< 0.01) than HFD group. In addition, the accumulation of visceral adipose tissues (*P* < 0.05) and liver fat (*P* < 0.05) were significantly inhibited in acupuncture group compared to HFD group. Nonetheless, there were no stark differences in the indices of adiposity and liver between HFD and acupuncture groups (Figures 1(a)–1(f)). Each group's rats were fed with the same amount of food in the beginning.  Figure 2 showed that acupuncture could significantly decrease the food intake compared with the HFD group both at week 4 and at week 8 (P < 0.05, P < 0.001). These results suggested that acupuncture can reduce food intake in HFD-induced abdominal obese rats. These results imply that acupuncture may play a beneficial role in reducing body weight and suppressing body fat accumulation, which also suggested that acupuncture could decrease appetite in HFD-induced abdominal obese rats.

### 3.2. Effects of Acupuncture on Metabolic Parameters

**Figure 3:**
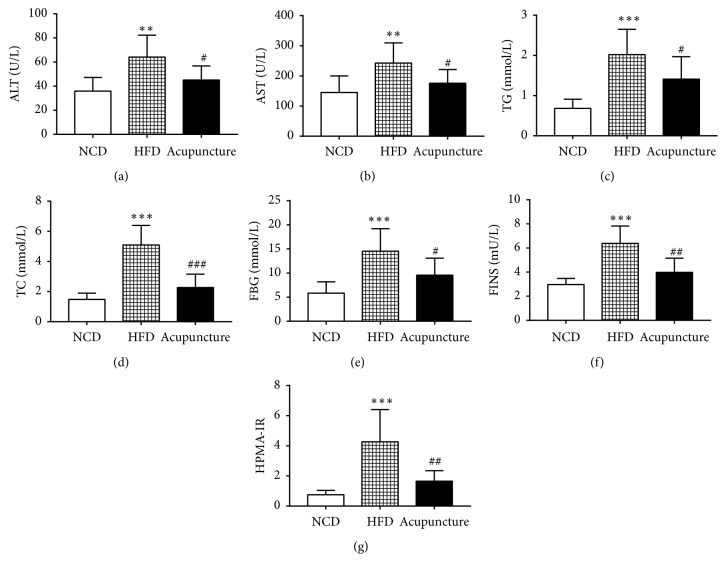
Effects of acupuncture on metabolic parameters at week 8. (a) Changes in alanine aminotransferase (ALT) among NCD, HFD, and acupuncture groups (n=7 in each group). (b) Changes in aspartate aminotransferase (AST) among the three groups. (c) Variation in triglycerides (TG). (d) Variation in total cholesterol (TC). (e) Levels of fasting blood glucose (FBG). (f) The serum levels of fasting insulin (FINS). (g) The index of homeostasis assessment of insulin resistance (HOMA-IR). All data are expressed as mean ± SD. ^∗^*P* < 0.05, ^∗∗^*P* < 0.01, ^∗∗∗^*P* < 0.001, NCD vs. HFD; ^#^*P* < 0.05, ^##^*P* < 0.01, HFD vs. acupuncture.

As shown in Figures 3(a)-3(b), the serum levels of ALT (*P* < 0.01) and AST (*P* < 0.01) were markedly increased in HFD group compared to NCD group. However, the levels of ALT (*P* < 0.05) and AST (*P* < 0.05) were significantly lower in acupuncture group than HFD in (*P* < 0.05). Besides, the serum lipids levels of TG (*P *< 0.001) and TC (*P *< 0.01) were significantly higher in HFD group compared to NCD group. Likewise, the significant results were found in the serum lipids levels of TG (*P* < 0.05) and TC (*P* < 0.05) between acupuncture and HFD groups (Figures 3(c)-3(d)). In addition, the level of FBG (*P* < 0.001) was significantly elevated in HFD group compared to NCD group. Notably, acupuncture treatment apparently decreased the level of FBG (*P* < 0.05) in HFD-induced abdominal obesity rats compared to nontreated HFD rats (Figure 3(e)). Furthermore, the levels of FINS and HOMA-IR were dramatically increased (*P* < 0.001) in HFD group compared with NCD group, while being significantly decreased (*P *< 0.01) in acupuncture group compared to HFD group (Figures 3(f)-3(g)). Taken altogether, these results indicate that acupuncture can inhibit the elevated blood lipid levels and improve insulin resistance in HFD-induced obese rats.

### 3.3. Influence of Acupuncture on the Diversity and Richness of Gut Microbiota

**Figure 4:**
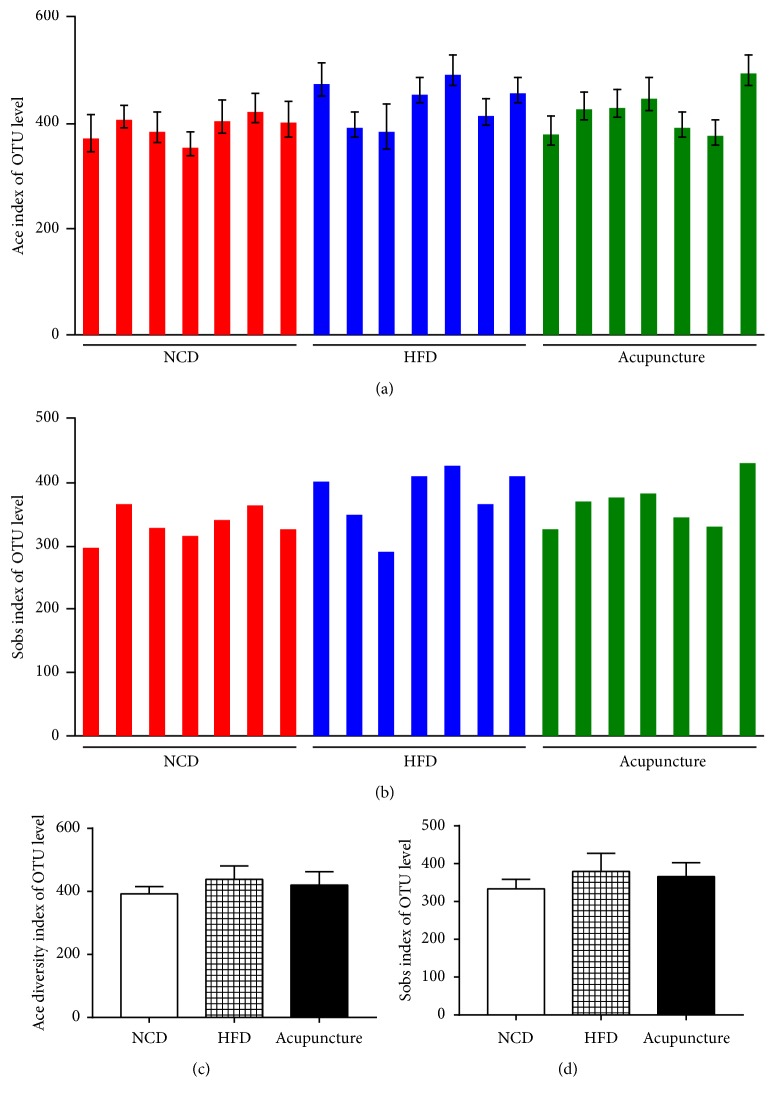
Influence of acupuncture on the diversity and richness of gut microbiota (n=7). (a-b) The alpha diversity of ace and sobs on the OTU levels. (c-d) Ace and sobs indexes. All data are expressed as mean ± SD. ^∗^*P* < 0.05, ^∗∗^*P* < 0.01, ^∗∗∗^*P* < 0.001, NCD vs. HFD; ^#^*P* < 0.05, ^##^*P* < 0.01, HFD vs. acupuncture.

In total of 941,816 high-quality 16S rRNA sequences were generated from 21 fecal samples (length distribution in 420-460 bp) and the average length of the sequence was 439 per each sample obtained (min: 328, max: 461). Each sample was normalized to an equal sequencing depth and clustering. After filtering the low abundant (< 0.1%) OTUs, 709 OTUs were obtained at ≥ 97% sequence identity. As shown in Figures 4(a)–4(d), the richness and diversity of ace and sobs indices were not significant differences among NCD, HFD, and acupuncture groups at the OUT level (Figure 4).

### 3.4. Impacts of Acupuncture on the Structure of Gut Microbiota

**Figure 5:**
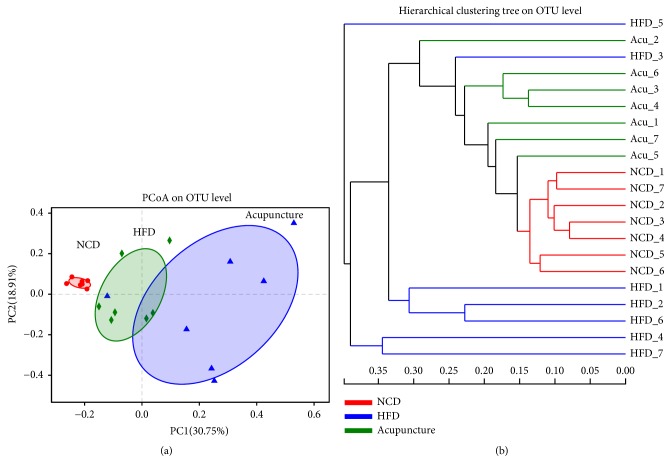
Responses of acupuncture on the structure of gut microbiota. (a) and (b): principal coordinate analysis (PCoA) on the OUT level. Sample hierarchical clustering results of the unweighted UniFrac distances of microbial 16S rRNA from the V3-V4 region, with multivariate analysis of variance (MANOVA).

To assess the similarities and differences in the composition of gut microbiota among NCD, HFD, and acupuncture groups, two aspects of beta diversity and UniFrac-based PCoA were examined. The results demonstrated an obvious clustering of gut microbiota in the three groups (Figure 5(a)). Notably, the composition of the gut microbiota in HFD group displayed an obvious shift that gathered separately from both NCD and acupuncture groups (Figure 5(a)). In addition, PCoA scores indicated that the gut microbiota of acupuncture group exhibited a movement in the first principal component (PC1) towards the direction of NCD group (Figure 5(a)). Hierarchical clustering analysis revealed that the microbial communities in HFD group were markedly different from NCD group. Moreover, the microbial communities in acupuncture group showed more similarities to those in NCD group (Figure 5(b)). In overall, acupuncture significantly reverted the HFD-induced variations along PC2 but prominently shifted the structure along PC1 (Figure 5), suggesting that the total gut microbial community is significantly altered by this treatment.

### 3.5. Modulation of the Composition of Gut Microbiota by Acupuncture at the Phylum Level

**Figure 6:**
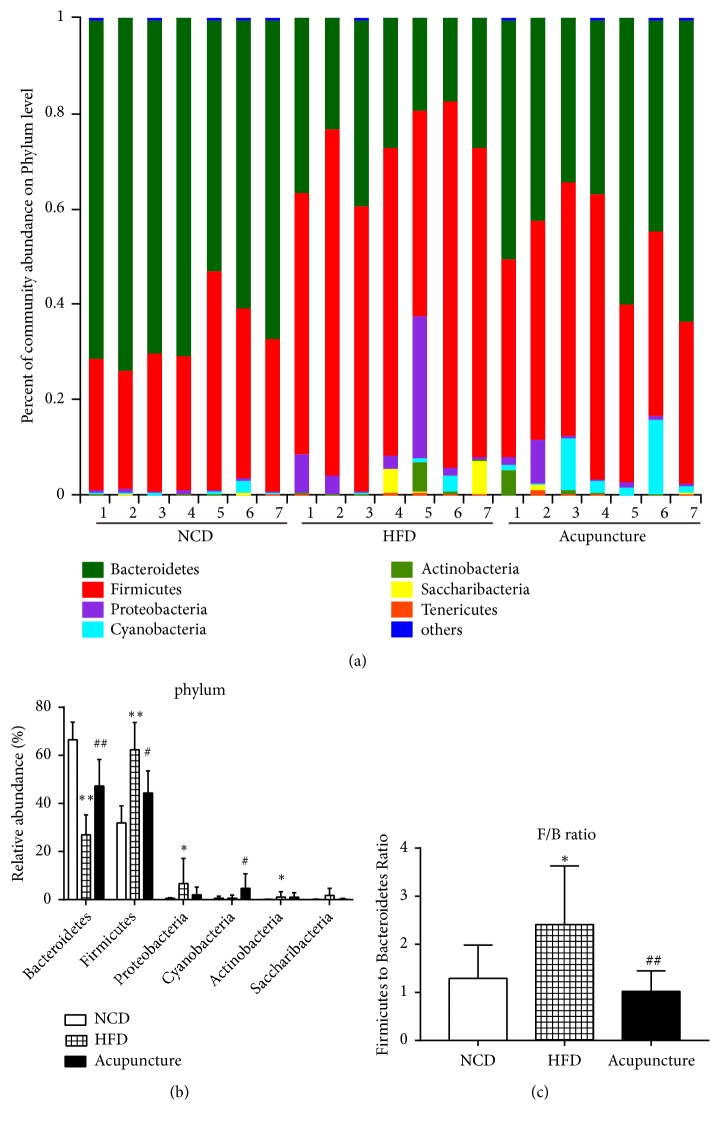
Modulation of the composition of gut microbiota by acupuncture at the phylum level. (a) The relative abundance of major taxonomic groups at the phylum level. (b) Significant differences of the relative abundance of gut microbiota at the phylum-level. (c) The Firmicutes/Bacteroidetes ratio in each group. All data are expressed as mean ± SD. ^∗^*P* < 0.05, ^∗∗^*P *< 0.01, ^∗∗∗^*P* < 0.001, NCD vs. HFD; ^#^*P* < 0.05, ^##^*P *< 0.01, HFD vs. acupuncture.

To determine the specific changes in gut microbiota, the relative abundance of major taxonomic groups at the phylum level was measured in NCD, HCD, and acupuncture groups. After taxon-based analysis, the most abundant phyla identified in the gut microbial compositions were Firmicutes and Bacteroidetes, while other phyla such as Cyanobacteria, Actinobacteria, and Saccharibacteria and a number of unclassified bacteria were presented in relatively lower abundance (Figure 6(a)). In particular, the relative compositions of Firmicutes constituted up to 32%, 59%, and 44% of the total bacterial sequences in NCD, HFD, and acupuncture groups, respectively (Figure 6(a)). Meanwhile, the relative compositions of Bacteroidetes were accounted for 67%, 30%, and 47% in NCD, HFD, and acupuncture groups, respectively (Figure 6(a)). There were significant dissimilarities in the relative abundances of the dominant phyla such as Firmicutes and Bacteroidetes among the three groups (Figure 6). Higher abundance of Firmicutes (*P* < 0.01) and reduced Bacteroidetes abundance (*P* < 0.01) were observed in HFD group compared to NCD group. Interestingly, 8 weeks of acupuncture treatment significantly increased the relative abundance of Bacteroidetes (*P* < 0.01) and decreased the relative abundance of Firmicutes (*P *< 0.05) compared to HFD group. Besides, among the low abundant phyla, the relative abundances of Proteobacteria and Actinobacteria were remarkably higher in HFD group than in NCD group (*P *< 0.05), and the abundance of Cyanobacteria was significantly greater in acupuncture group compared to HFD group (*P *< 0.05). However, there were no differences in the relative abundance of phylum Saccharibacteria among the three groups (Figure 6(b)). In addition, compared to NCD group, the ratio of Firmicutes to Bacteroidetes was significantly increased in HFD group (*P *< 0.05) and reduced in acupuncture group (*P* < 0.01) (Figure 6(c)). These results indicate that acupuncture can regulate the balance of gut microbiota in HFD-induced abdominal obese rats at the phylum level, by adjusting the relative abundances of major phyla Firmicutes and Bacteroidetes.

### 3.6. Alterations of the Composition of Gut Microbiota following Acupuncture at the Genus Level

**Figure 7:**
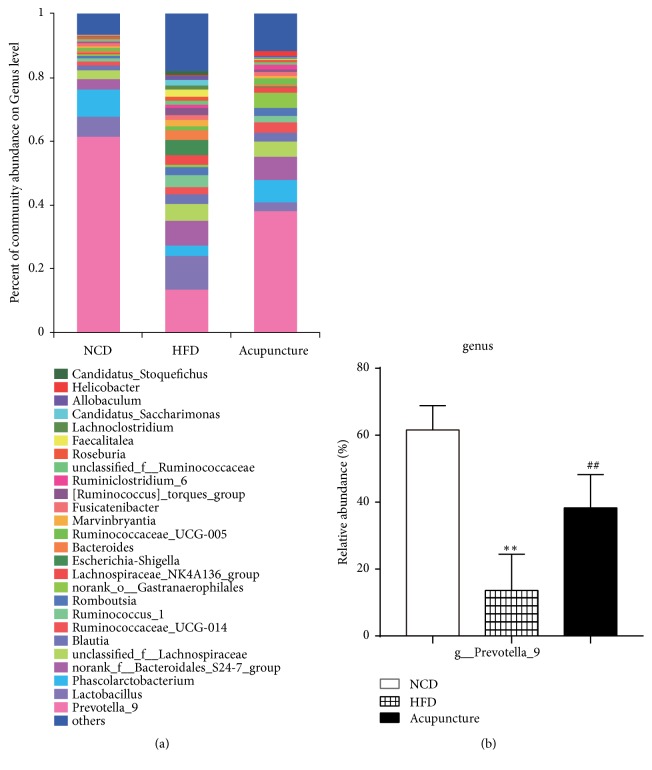
Alterations of the composition of gut microbiota following acupuncture at the genus level. (a) The relative abundance of gut microbiota at the genus level. (b) Significant differences of the main genus Prevotella_9. All data are expressed as mean ± SD. ^∗^*P* < 0.05, ^∗∗^*P *< 0.01, ^∗∗∗^*P* < 0.001, NCD vs. HFD; ^#^*P* < 0.05, ^##^*P* < 0.01, HFD vs. acupuncture.

To explore the deeper taxonomic composition of microbial communities, the abundances of gut microbiota were analyzed the genus level. As shown in Figure 7(a), Prevotella_9 dominated the taxonomic distribution of gut microbiota among the three groups. Notably, the relative abundance of genus Prevotella_9 was significantly decreased in HFD group compared to NCD group (*P *< 0.01), and treatment with acupuncture (*P *< 0.01) dramatically increased the abundance of Prevotella_9 (Figure 7(b)). These results suggest that acupuncture is beneficial and can reduce abdominal obesity. Furthermore the acupuncture group of rats has increased levels of Prevotella_9, which maybe suggests that this microbial group is related to the lean phenotype as it is also highly abundant in the NCD group. Interestingly, acupuncture can modulate the composition of gut microbiota by altering the relative abundance of Prevotella_9 at the genus level.

## 4. Discussion

In the present study, acupuncture treatment of GB 26 acupoint significantly decreased the body weight, WC, and visceral adipose tissues, reduced the food intake, and enhanced insulin sensitivity, glucose homeostasis, and lipid metabolism in abdominal obese rats. Moreover, acupuncture significantly reduced Firmicutes abundance, elevated Bacteroidetes abundance, and lowered the ratio of Firmicutes to Bacteroidetes. Additionally, the relative abundance of Prevotella_9 was significantly increased by acupuncture treatment at the genus level. These findings strongly indicate that acupuncture can suppress abdominal fat accumulation, regulate hepatic glucose and lipid metabolism, ameliorate insulin resistance, and exert antiobesity effects via the regulation of gut microbiota, which also suggested that acupuncture can suppress appetite.

Accumulating data have demonstrated that the alteration of gut microbiota is associated with obesity in both humans and animals [33–36]. Besides, acupuncture treatment exerts beneficial effect on obesity by alternating the composition of gut microbiota [37]. However, previous studies have demonstrated conflicting results on the association between Firmicutes/Bacteroidetes ratio and the risk of obesity [38], [39–41]. Some studies have reported that the imbalance of gut microbiota, with an increase in Firmicutes and a corresponding decrease in Bacteroidetes, is an independent risk factor for obesity [42, 43]. However, other studies have negated the importance of Firmicutes/Bacteroidetes ratio as a crucial factor that influences obesity [44, 45]. In this study, there were no significant differences in the gut microbial community diversity among NCD, HFD, and acupuncture groups. The abdominal obese rats in HFD group exhibited a higher ratio of Firmicutes/Bacteroidetes, which is consistent with previous findings [46, 47]. At the genus level, the proportion of Prevotella_9 was significantly increased in acupuncture-treated abdominal obese rats. Therefore, these gut microbial changes may indicate the important role of acupuncture treatment in abdominal obesity.

With regard to the mechanisms underlying the antiobesity effects of acupuncture treatment on gut microbiota structure, it is yet to be clarified whether those gut microbiota variations are causal risk factors of obesity or simply reflect the acupuncture treatment. Most of the researchers believed that their relationship is dependent on the theory of brain-gut axis [48, 49]. In addition, gut microbiota can regulate food intake and energy balance, which is crucial for the development of obesity [50]. The changes in gut microbiota may directly or indirectly target the brain through vagal stimulation or immune-neuroendocrine mechanism, respectively [51, 52]. Current studies have showed that acupuncture reduced food intake and body weight by regulating the appetite-related hormones section in the hypothalamus, including neuropeptide Y (NPY), cocaine-and amphetamine-regulated transcript (CART), and proopiomelanocortin (PoMC) [53, 54]. Acupuncture can activate and release brain-gut peptide, followed by mediating gastrointestinal motility and intestinal flora [55]. Collectively, these findings suggest a potential role of brain-gut axis in controlling body weight, decreasing appetite, and regulating glucose and lipid metabolism during acupuncture treatment. Further studies are warranted to reveal the exact mechanisms underlying the effects of acupuncture on the modulation of gut microbiota and food intake, thereby participating in regulating body weight control and lipid metabolism in abdominal obesity.

## 5. Conclusions

The present study indicates that acupuncture can effectively ameliorate abdominal obesity, decrease appetite, improve metabolic parameters, and modify gut microbiota composition. Acupuncture can lower Firmicutes/Bacteroidetes ratio at the phylum level and enhance the abundance of Prevotella_9 at the genus level. These alterations in gut microbiota may serve as a novel mechanism underlying the therapeutic effects of acupuncture on abdominal obesity. Nevertheless, more research is needed to evaluate the complex interactions among acupuncture treatment, gut microbiota dysbiosis, and abdominal fat accumulation, before their clinical implications in human obesity.

## Data Availability

The data used to support the findings of this study are available from the corresponding author upon request.

## Supplementary Materials

Supplementary Materials (1) LEfSe analysis of each group. (2) Data of principal coordinates analysis (Pcoa) in each sample at the OTU level. (3) Eigen values of Pcoa from PC1-PC21. (4) Statistical data of each group at the genus level. (5) Statistical data of each group at the phylum level.
